# Predictors of durability of the prostatic urethral lift (PUL) for benign prostatic hyperplasia

**DOI:** 10.1038/s41391-026-01101-y

**Published:** 2026-05-06

**Authors:** Gregg Eure, Matt Ashley, Daniel B. Rukstalis, Peter Chin, Neil Barber, Claus Roehrborn

**Affiliations:** 1 Urology of Virginia, Culpeper, VA USA; 2 Summit Health, Bend, OR USA; 3 Carilion Clinic, Roanoke, VA USA; 4 South Coast Urology, Wollongong, NSW Australia; 5 Frimley Park Hospital, Frimley, UK; 6 UT Southwestern, Dallas, TX USA

**Keywords:** Outcomes research, Prostatic diseases, Medical research

## Abstract

**Background:**

There is a need to understand the predictors of prostatic urethral lift (PUL) durability to help physicians counsel patients and optimize outcomes.

**Methods:**

A simple logistic regression analysis was performed on a pooled dataset of 331 subjects across 5 PUL controlled studies with up to 5 years follow up. Predictors of “surgical durability” (no surgical treatment for return of symptoms) and “total durability” (no surgical or medical treatment for return of symptoms) were assessed at 1- and 5-year post-procedure.

**Results:**

Placing ≤4 implants regardless of baseline prostate volume was associated with increased surgical retreatment within the first year. Through one year, more implants were protective of retreatment; for every incremental implant placed, surgical retreatment odds decreased an additional 41.2% and risk of combined surgical or medical retreatment decreased 30.2%. Higher implant density was also protective through one year; for each additional implant placed per 10cc prostate volume, surgical retreatment odds decreased incrementally by 79.0%. Up to 5 years, a higher baseline pretreatment IPSS score predicted higher rates of surgical or medical retreatment; for every one-point score increase of baseline IPSS, surgical or medical retreatment odds increased 5.2%. Baseline demographics, medical history and baseline sexual function were not predictors of surgical or total durability.

**Conclusions:**

More implants, higher implant density, and lower baseline IPSS are all associated with decreased surgical and medical retreatment rates up to 5 years following PUL. These findings inform optimizing PUL technique, considering earlier intervention and counseling men on outcomes prior to PUL.

## Introduction

Benign prostatic hyperplasia (BPH) is a common condition among aging men for which there are many treatment options. Clinicians should evaluate treatment options in a shared decision-making process with patients [1], which includes understanding the criteria that yield the highest likelihood of success for a given patient and therapy [2]. The American Urological Association (AUA) guideline on the management of lower urinary tract symptoms (LUTS) attributable to BPH emphasizes durability of effect as an important factor to consider [2]. The prostatic urethral lift (PUL) has been proven to deliver rapid, meaningful and durable symptom relief with minimal morbidity [3–8]. The durability of PUL treatment is well-understood with surgical retreatment rates of 11.6–13.7% by 5 years post-procedure as reported in real-world and controlled studies [3, 9]. While overall retreatment rates have been thoroughly documented and consistent throughout the literature, predictors of what may predispose some men to lower or higher rates as compared to the mean have not been well-understood. Appreciating the predictors of medical and surgical durability could help clinicians better understand how to optimize outcomes and counsel patients.

## Methods

A univariable logistic regression analysis was performed on a pooled dataset across 5 PUL controlled studies with variable follow-up ranging up to 5 years post-procedure. “Surgical durability” was defined as no surgical retreatment (i.e., a second BPH procedure including a repeat PUL or any other BPH surgery available at the time of the study) for BPH during the defined follow-up period. The definition of surgical durability does not take into consideration the use of medications for BPH or lower urinary tract symptoms (LUTS). “Total durability” was defined as no surgical or medical treatment (alpha blocker, 5 ARI, anticholinergic [Supplementary Table 1]) for BPH/LUTS following PUL during the defined follow-up period. Durability was examined at 1- and 5-year post-procedure with these follow up times chosen based on availability of data in the pooled dataset (Supplementary Fig. 1). Additional baseline data analyzed as potential predictors of durability included baseline demographics (e.g. race, BMI, prostate volume, PSA), symptom status (e.g. IPSS total score and domain scores), medical history (e.g. history of urinary retention, urinary tract infection, BMI, history of cancer) and measures of sexual function (e.g. MSHQ, SHIM). Procedural data including number of implants placed and subsequent implant density (based on prostate size and noted as implants per 10cc of tissue) were also recorded. All analyses were reported in their unadjusted form with p-values.

## Results

**Fig. 1:**
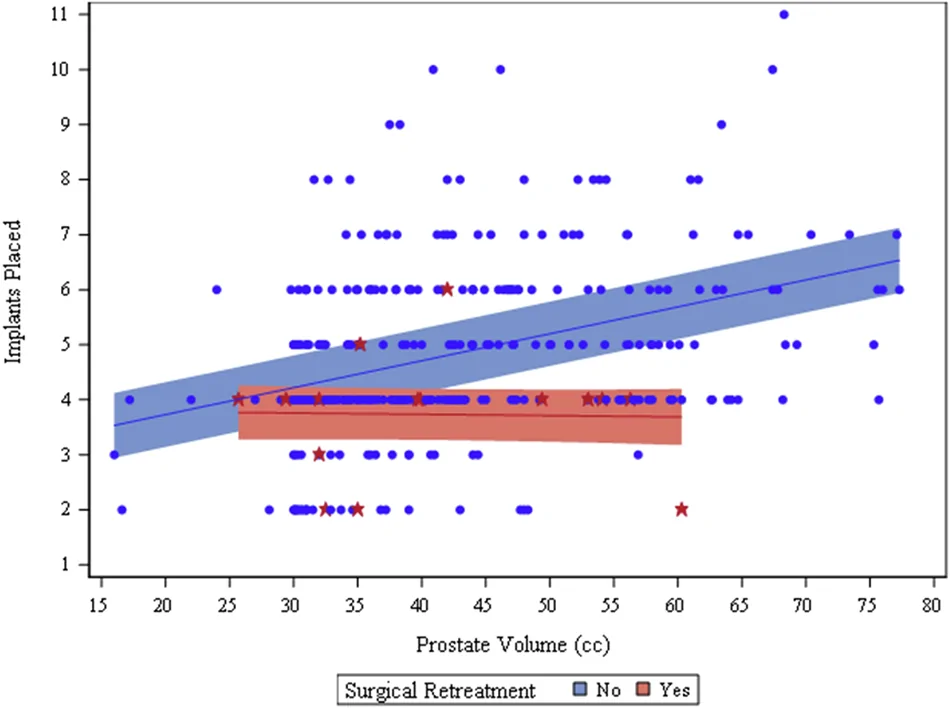
Regression analysis of the number of implants placed vs baseline prostate volume for subjects with and without surgical retreatment through 1 year. Best fit lines with confidence bands are depicted.

The pooled dataset included 331 subjects (Table 1). The average number of implants was 4.8 ± 1.6 with more implants placed with increasing prostate volume on average (Fig. 1). Placing 4 or fewer implants regardless of baseline prostate volume was associated with a higher rate of surgical retreatment, while no subject with ≥7 implants placed was surgically retreated within 1 year (Fig. 1).

**Table 1:** Prostatic urethral lift (PUL) controlled studies included.

Prospective trial design	Randomized, PUL vs. sham	Randomized, PUL vs. TURP	Single arm, in office, local anesthesia	Single arm, obstructive middle lobe included	Crossover from sham in L.I.F.T. study	Pooled analysis of LIFT, BPH6, LOCAL, MedLift and crossover
Subjects (*N*)	140	44	51	45	51	331
Age	67	63.8	65.5	63.9	64	65.5
PSA	2.4	2.5	1.8	2.5	2.2	2.3
Prostate volume	44.5	37.8	41.3	44.2	40.5	42.5
IPSS	22.2	22.1	21.5	24.2	25.4	22.8
PVR	85.5	85.9	77.1	107.3	88.1	87.6
Qmax	7.9	9.2	8.2	7.2	8	8
Number of implants	4.9	4.7	3.7	6.3 (1.3 for median lobe)	4.5	4.8

### One-year durability

Through one year, an increasing number of implants was a predictor of both surgical and total durability; for every incremental implant placed, the odds of surgical retreatment were reduced by 41.2% and the odds of total retreatment were reduced by 30.2%. Though prostate volume alone was not a significant predictor of durability, a higher implant ratio (number of implants per 10cc prostate volume) did significantly predict higher rates of surgical durability (Table 2); for each additional implant placed per 10cc prostate volume, the odds of surgical retreatment were reduced by 79.0%.

**Table 2:** Logistic regression model for predictors of surgical durability and total durability through 1 year.

	1-year		1-year	
	Surgical retreatment		Total retreatment	
Baseline feature	Odds ratio	*p*-value	Odds ratio	*p*-value
Number of implants placed	0.588	0.0097	0.698	0.023
Prostate volume	0.989	0.65	0.995	0.78
Implant ratio (implants per 10cc prostate volume)	0.21	0.049	0.33	0.068
PSA	0.923	0.62	1.068	0.54
Procedure time	0.973	0.28	0.99	0.52
IPSS total score	1.027	0.58	1.035	0.38
Age^a^	0.992	0.8	1.006	0.828
History of UTI	1.039	0.961	1.71	0.608
History of prostatitis	1.058	0.957	1.71	0.608
History of prostate cancer	0.515	0.402	0.856	0.841
BMI	1.021	0.719	0.999	0.988

^a^Age is calculated here as a hazard ratio from cox proportional hazard model.

### Five-year durability

Within a 5-year follow up period, a higher baseline pretreatment IPSS score was a predictor of higher rates of total retreatment; for every one-point increase in baseline pretreatment IPSS score, the odds of total retreatment were increased incrementally by 5.2% (Table 3). Other baseline demographics, medical history and baseline sexual function were not predictors of surgical or total durability.

**Table 3:** Logistic regression model for predictors of surgical durability and total durability through 5 years.

	5-year		5-year	
	Surgical retreatment		Total retreatment	
Baseline feature	Odds ratio	*p*-value	Odds ratio	*p*-value
Number of implants placed	0.949	0.64	1.037	0.69
Prostate volume	1.004	0.76	1.012	0.32
Implant ratio (implants per 10cc prostate volume)	1.038	0.3706	1.033	0.3514
PSA	1.089	0.31	1.148	0.058
Procedure time	0.983	0.17	1.002	0.84
IPSS total score	1.046	0.14	1.052	0.045
Age^a^	1.009	0.53	1.018	0.087
History of UTI	1.175	0.756	0.746	0.458
History of prostatitis	3.379	0.245	1.43	0.547
History of prostate cancer	1.228	0.753	1.096	0.857
BMI	0.996	0.905	1.006	0.835

^a^Age is calculated here as a hazard ratio from cox proportional hazard model.

## Discussion

This first-ever analysis of the predictors of medical and surgical durability after PUL demonstrates that a higher total number of implants placed and increased implant density are predictors of decreased retreatment risk. The findings are consistent with the known secondary and long-term mechanism of action of PUL implants: mechanical compression leading to relative ischemia, ultimately causing tissue necrosis and remodeling of the prostate gland [10].

The other patient factor that correlated with decreased risk of surgical or medical retreatment over 5 years was a lower baseline pre-treatment IPSS score. This suggests that intervening with PUL earlier in the BPH disease process may possibly lower the risk of that patient requiring medical or surgical retreatment. This proposed protective benefit of early procedural intervention has been suggested in a handful of other BPH publications, including that by Flanigan et al. comparing outcomes of men undergoing immediate versus delayed TURP [11]. There are several hypotheses for why higher baseline IPSS scores predict higher rates of retreatment. Namely, there is a suspicion that these men have sustained more severe and less reversible damage to their detrusor function, which results in less symptomatic improvement after the index procedure and higher likelihood of future medical or procedural intervention [12, 13].

It is also important to note that the predictors of durability through one year were different from those through 5 years – one-year predictors of durability included number of implants placed and implant density, whereas 5-year durability was predicted by baseline pretreatment IPSS score. The implant number and density predictors seen earlier in the postoperative experience may suggest that the consequences of inadequate compression manifest themselves earlier through a suboptimal initial treatment response. Predictors of retreatment associated with more severe baseline urinary symptoms, and presumed underlying detrusor dysfunction, may have a slower and more insidious progression of symptom recurrence, which may result in more delayed treatment. More studies are needed to explore these hypotheses.

This analysis has practical applications to help clinicians counsel patients and optimize the longevity of outcomes. The results suggest that surgeons may successfully treat larger prostates with durable results provided an adequate number of implants is used. In this univariable analysis, placement of four or fewer implants across a range of prostate volumes was associated with higher retreatment rates, whereas use of more than four implants and higher implant density (>1 implant per 10cc of prostate tissue) appeared to be associated with lower retreatment rates. Patients should be counseled that receiving treatment while symptoms are milder significantly decreased the odds of surgical or medical retreatment in this univariable analysis; conversely, patients with higher IPSS scores were associated with a higher risk of retreatment 5 years post-procedure. However, it should be noted that, though this analysis can demonstrate signals for increased risk of retreatment, the treating physician should apply the appropriate number of implants based on their experience and discretion, rather than applying a standard number of implants to every patient.

Notable patient factors that did not independently affect medical or surgical durability included median lobe anatomy, baseline maximum flow rate, and prostate size.

The study was limited by only including controlled PUL studies, all of which were sponsored by Teleflex, and the lack of intermediate data between 1 and 5 years; the latter could have revealed other predictors of durability as well as whether the number or density of implants affect durability beyond one year and whether total baseline IPSS score affects durability out to 2–4 years. Additionally, cases were not controlled for setting of PUL (i.e., hospital inpatient, outpatient or ambulatory surgery center); given the variability of implant reimbursement in different settings, this information could have provided useful information. It is also important to note that the statistical analysis performed on this pooled dataset was univariable logistic regression. Therefore, the impact of each variable was only assessed individually without controlling for other factors, and the results may be interpreted as hypotheses that require further investigation. While a multivariable logistic regression analysis would be desirable when assessing the impact of multiple factors, in this case, the outcomes of interest occurred in too few patients to successfully perform a multivariable logistic regression. These observations should be viewed as hypothesis-generating rather than definitive, and further studies controlling for procedure setting, increasing patient numbers for multivariable logistic regression, and including real-world data are needed to better understand patient- and procedure-level predictors of durability. Importantly, implant number should continue to be individualized based on prostate anatomy, symptom profile, and physician judgment rather than adherence to a fixed numerical threshold.

## Conclusions

Ensuring an adequate number of implants (greater than 4 regardless of prostate size) and sufficient implant density (at least 1 per 10cc of tissue) are significant factors in predicting lower risk for surgical and medical retreatment after PUL. Furthermore, treating patients with lower IPSS scores at the time of PUL may minimize risk for retreatment at 5 years post-procedure.

## Supplementary information

Supplemental Material - Figure and Table Legends

Supplemental Figure 1

Supplemental Table 1

## Data Availability

The datasets generated and/or analyzed during the current study are available from the corresponding author on reasonable request.
