# Supplementary material for: Predictors of durability of the prostatic urethral lift (PUL) for benign prostatic hyperplasia
Source: Prostate Cancer Prostatic Dis. 2026 May 6;29(4):836–9. doi: 10.1038/s41391-026-01101-y (PMC13623641; doi:10.1038/s41391-026-01101-y)
Supplement: Supplementary file 1 [file 41391_2026_1101_MOESM1_ESM.docx]

Supplemental Information – figure and table legends

Supplemental Figure 1 – Product-Limit Failure Curve of Pooled Studies through 5 Years

Supplemental Table 1 – Details of Each Study in Pooled Analysis
